# Optimization of the Purification Process for *Lactiplantibacillus plantarum* Lipoteichoic Acid with Anti-Biofilm Properties against Dental Pathogens

**DOI:** 10.4014/jmb.2506.06045

**Published:** 2025-09-22

**Authors:** Jeongmin Shin, Dong Hyun Park, Woohyung Jun, Ok-Jin Park, Cheol-Heui Yun, Jintaek Im, Seung Hyun Han

**Affiliations:** 1 Department of Oral Microbiology and Immunology, and Dental Research Institute, School of Dentistry, Seoul National University, Seoul 08826, Republic of Korea; 2 Department of Agricultural Biotechnology, and Research Institute of Agriculture and Life Sciences, Seoul National University, Seoul 08826, Republic of Korea; 3 Institutes of Green-bio Science and Technology, Seoul National University, Pyeongchang, Gangwon-do 25354, Republic of Korea

**Keywords:** Lipoteichoic acid, *Lactiplantibacillus plantarum*, oral pathogens, anti-biofilm activity, LTA intermediates

## Abstract

Biofilms formed by oral pathogens play a critical role in the initiation and development of various dental diseases by enhancing resistance to dental medicaments. Previously, we reported the potent anti-biofilm activity of *Lactobacillus* lipoteichoic acid (LTA), a major cell wall component of Gram-positive bacteria, against various dental pathogens. Nevertheless, the practical application of LTA as an anti-biofilm agent is limited due to its complex, cost- and labor-intensive purification processes. Therefore, to minimize the purification processes required to obtain LTA with anti-biofilm activity, we isolated LTA from *Lactiplantibacillus plantarum* (Lp.LTA) and its intermediates following each purification step, and compared their anti-biofilm activity against dental pathogens. The Lp.LTA intermediates underwent sequential purification after butanol extraction and hydrophobic-interaction chromatography, and these were designated as LTA-Butanol and LTA-HIC, respectively. The results of Western blot analysis demonstrated that LTA-HIC has a higher concentration of LTA than LTA-Butanol. Although both LTA-Butanol and LTA-HIC dose-dependently inhibited *Streptococcus mutans* and *Porphyromonas gingivalis* biofilm formation, the anti-biofilm activity of LTA-HIC was superior to that of LTA-Butanol and comparable to Lp.LTA. Furthermore, sodium hydroxide treatment of LTA-HIC diminished its anti-biofilm activity, suggesting that LTA is a key component responsible for the anti-biofilm capacity of LTA-HIC. Collectively, these results demonstrated that LTA-HIC serves as an intermediate in the LTA purification, retaining its anti-biofilm properties and offering a viable solution to the challenges associated with the LTA purification process.

## Introduction

Bacterial biofilms are aggregates of microorganisms attached to a surface of diverse biotic and abiotic materials [1]. In general, biofilm formation initiates from the surface attachment of planktonic bacteria, and the adhered bacteria then produce an exopolysaccharide (EPS), subsequently leading to formation of a multi-layered EPS matrix consisting of proteins, polysaccharides, and extracellular DNA [2]. The multi-layered EPS matrix permits bacterial aggregation within the biofilm and creates a physical barrier, resulting in increased resistance to anti-microbial agents compared to their planktonic state [3, 4]. Moreover, it has been reported that the bacteria within biofilms can readily evade host immune responses, such as phagocytosis by macrophages or neutrophils [5, 6]. Bacterial biofilms are regarded as a major public health concern that limits medical treatment alternatives to control bacterial infections.

Dental caries and periodontitis are among the most prevalent and severe oral diseases, resulting from infections by dental pathogens, ultimately leading to tooth loss [7, 8]. Moreover, *Streptococcus mutans*, a facultative anaerobic Gram-positive bacterium, is considered as a major cariogenic pathogen predominantly detected in the oral cavity of patients with dental caries [1] while *Porphyromonas gingivalis*, a strict anaerobic Gram-negative bacterium, is regarded as a keystone periodontopathogen frequently found in chronic periodontitis patients [9]. Accumulating reports have demonstrated that these dental pathogens can initiate and/or get worse the severity of dental caries and periodontitis via their biofilm formation, which offers increased resistance to anti-microbial medications, and host immune responses [1, 10, 11]. Currently available dental medicaments, including disinfectants and antibiotics, have shown limited effectiveness against biofilms formed by these dental pathogens [12]. Moreover, the prolonged application of the previously mentioned dental treatments is often accompanied by various side effects, including soft-tissue lesions, taste alteration, and allergic reactions [13, 14]. Therefore, the development of alternative strategies is indispensable for managing dental pathogen biofilms with marginal adverse effects.

Probiotics, particularly *Lactobacillus* spp., serve as effective anti-biofilm reagents against various dental pathogens without adverse effects. Previous studies reported that *Lactiplantibacillus plantarum*, *Limosilactobacillus reuteri*, and *Lacticaseibacillus rhamnosus* GG suppressed the biofilm formation of *S. mutans* and/or *P. gingivalis* [15, 16]. The anti-biofilm activity of *Lactobacillus*-derived biomolecules against dental pathogens has also been demonstrated. *Lactobacillus*-derived biosurfactants potently inhibited the *Candida albicans* biofilms [17], while *L. plantarum* culture supernatant efficiently suppressed *S. mutans* biofilm formation [18]. Additionally, reuterin purified from *L. reuteri* reduced the biofilms of *P. gingivalis* and *Treponema denticola* [19].

Lipoteichoic acid (LTA) is a representative cell wall component of Gram-positive bacteria, which is involved in bacterial growth, cell wall integrity, adhesion, biofilm formation, and evasion of host immune responses [20-22]. LTA consists of an amphiphilic glycolipid linked to a hydrophilic polyphosphate polymer. Based on bacterial species, LTAs are characterized into five structurally distinct types, ranging from types I to V [21]. In addition, the LTAs derived from *Lactobacillus* spp. have a poly-glycerol phosphate (Gro-P) backbone containing D-alanine, glucose, or galactose residues, linked with a triacylated glycolipid moiety [23]. Recently, we reported that LTAs purified from *Lactobacillus* spp. potently inhibited biofilm formation and/or disrupted the pre-formed biofilm of dental pathogens, such as *S. mutans*, *Streptococcus gordonii*, and *Enterococcus faecalis* [24-27], suggesting that LTA is a major *Lactobacillus*-derived molecule responsible for their anti-biofilm capacity. Furthermore, our previous studies demonstrated that *L. plantarum* LTA (Lp.LTA) has the most potent anti-biofilm activity against the previously mentioned dental pathogens among the tested LTAs from *Lactobacillus* spp. [24, 25].

Despite the superior anti-biofilm capacity of Lp.LTA, the complex purification process involving multiple steps of butanol extraction, hydrophobic-interaction chromatography, and anion-exchange chromatography limited its practical application as an anti-biofilm agent against oral pathogens. The complexity of LTA purification may restrict large scale isolation, suggesting a necessity for the minimization of the purification process. This study compares the anti-biofilm activities of Lp.LTA and its intermediates after each purification process to identify which intermediate retains the anti-biofilm properties of Lp.LTA, thereby optimizing the purification processes to obtain LTA with anti-biofilm activity.

## Materials and Methods

### Reagents and Chemicals

Brain heart infusion (BHI) and Bacto agar were purchased from BD biosciences (USA), while De Man, Rogosa, and Sharpe (MRS) was obtained from KisanBio (Republic of Korea). Hemin, menadione, Octyl-Sepharose CL-4B, DEAE-Sepharose DFF100, and proteinase K were purchased from Sigma-Aldrich (USA). Sulfuric acid, nitric acid, sodium hydroxide, *n*-butanol, and *n*-propanol were obtained from Junsei (Japan). All other chemicals were purchased from Sigma-Aldrich unless otherwise indicated.

### Bacteria and Culture Conditions

For the preparation of LTA and its intermediates, *L. plantarum* KCTC 10887BP was obtained from the Korean Collection for Type Culture (KCTC, Republic of Korea) and grown in MRS broth at 37°C under aerobic and static conditions. On the other hand, *S. mutans* UA159 and *P. gingivalis* ATCC 33277 were obtained from the American Type Culture Collection (ATCC, USA) for evaluating the regulatory effect of Lp.LTA and its intermediates on the biofilm formation of oral pathogens. *S. mutans* was grown in BHI broth at 37°C under aerobic and static conditions, while *P. gingivalis* was cultured in BHI broth containing 5 mg/ml of hemin and menadione at 37°C under anaerobic and static culture conditions using an anaerobic workstation (Whitley DG250, Don Whitley Scientific, UK).

### Preparation of LTA and Its Intermediates Depending on Purification Process

Lp.LTA and its intermediates after each purification process were prepared as previously described with minor modifications [28, 29]. Total 86 g of *L. plantarum* was initially harvested by centrifugation and then disrupted in 0.1 M sodium citrate buffer (pH 4.7) by ultrasonication (Vibracell VCX500, Sonics and materials, USA) for 90 min (30 s on and 20 s off cycles) on ice. An equal volume of *n*-butanol was added to the bacterial lysate and was centrifuged. After the centrifugation, the lower phase of butanol mixture was collected and dialyzed in endotoxin-free distilled water (Daihan Pharm. Co. Ltd, Republic of Korea) using a semi-permeable dialysis membrane (Spectrum Laboratories, USA). A part of the dialyzed lower phase of butanol mixture was lyophilized and designated as the “LTA-Butanol”. The remaining dialyzed lower phase of butanol mixture was further equilibrated with 15% *n*-propanol in 0.1 M sodium acetate buffer and was applied to hydrophobic-interaction chromatography using a column filled with Octyl-Sepharose CL-4B. After removing unbound materials with 15%*n*-propanol in 0.1 M sodium acetate buffer, the bound materials were eluted with 35% *n*-propanol in 0.1 M sodium acetate buffer and collected sequentially using a fraction collector (Bio-Rad, USA). Based on the results of inorganic phosphate assay, the aliquots containing phosphate were pooled and dialyzed under the previously mentioned conditions. A part of the dialysate was then lyophilized and designated as the “LTA-HIC”. The remaining dialysate was equilibrated with 30% *n*-propanol in 0.1 M sodium acetate buffer and applied to anion-exchange chromatography using a column filled with DEAE-Sepharose DFF100. Fractions were then eluted with a linear salt gradient (0 to 1 M NaCl in the equilibration buffer) and collected using a fraction collector. After the inorganic phosphate assay for the eluted DEAE-column fractions, the fractions were pooled and dialyzed. The dialysate was then lyophilized and designated as “Lp.LTA”. The quantities of LTA-Butanol, LTA-HIC, and Lp.LTA were determined by measuring their dry weight and were dissolved in endotoxin-free distilled water. To evaluate the purity of Lp.LTA and its intermediates, possible impurities, such as proteins, nucleic acids, and endotoxins, were examined in the Lp.LTA and its intermediates using bicinchoninic acid (BCA) protein assay kit (Thermo-Fisher Scientific, USA), spectrophotometer (Nano Drop 2000; Thermo-Fisher Scientific), and *Limulus* amoebocyte lysate assay kit (Lonza, Swiss), respectively.

### Inorganic Phosphate Assay

To identify the LTA-containing fractions after hydrophobic-interaction chromatography and anion-exchange chromatography, an inorganic phosphate assay was conducted as previously described [27]. Briefly, each fraction was blended with sulfuric acid and nitric acid and boiled in a glass tube. The mixture was then blended with 3 M sodium hydroxide, 100 mM molybdate in 5 M sulfuric acid, and 130 mM stannous chloride in glycerol, and its optical density was measured at 600 nm using a microplate reader (SPARK, TECAN, Swiss). Serially diluted potassium phosphate solution (Sigma-Aldrich) ranging from 2.5 to 320 μM was used as a standard for determining phosphate content of the fractions.

### Western Blot Analysis

Western blot analysis for LTA was performed as previously described [30, 31]. Briefly, various concentrations of LTA-Butanol, LTA-HIC, and Lp.LTA were subjected to 15% sodium dodecyl sulfate-polyacrylamide gel and then electro-transferred to a PVDF membrane (Millipore Sigma, USA) using a tank transfer system (Bio-Rad). The membrane was then washed with Tris-buffered saline containing Tween 20 (TBST; 20 mM Tris·HCl, 150 mM NaCl, and 0.05% Tween 20) and incubated in TBST containing 5% skim milk for 1 h. The membrane was washed with TBST twice and incubated with an antibody specific for Gro-P region of LTA (Hycult, Netherlands) at 4°C overnight. After washing a membrane with TBST, it was additionally incubated with HRP-conjugated anti-mouse IgG (Jackson Immuno Research, USA) at room temperature for 1 h. The immuno-reactive bands on the membrane were detected using ECL reagents (Dain Biotech, Republic of Korea) and visualized by a Vilber bio-image analyzer (Fusion FX6.0, Vilber, France). The LTA concentrations of LTA-Butanol and LTA-HIC were determined using the regression obtained from the band intensity of various concentrations of Lp.LTA using Image J Software (National Institutes of Health, USA).

### Enzyme-Linked Immunosorbent Assay (ELISA)

To evaluate the concentrations of LTA in LTA-Butanol and LTA-HIC, ELISA was conducted as previously described with minor modifications [32]. Briefly, a 96-well plate was incubated with high density lipoprotein (5 μg/100 μl/well, Sigma-Aldrich) at room temperature for 2 h. After the incubation, the plate was washed with phosphate buffered saline (PBS) containing 0.5% bovine serum albumin (BSA) and then incubated with PBS containing 2% BSA at 37°C for 1 h. The plate was washed with PBS containing 0.5% BSA three times and incubated with serially diluted LTA-Butanol, LTA-HIC, or Lp.LTA at 37°C for 2 h. After the incubation, the plate was sequentially incubated with an antibody specific for Gro-P region of LTA (Hycult), and a HRP-conjugated anti-mouse IgG (SouthernBiotech, USA) at room temperature for 1.5 h. After washing, the plate was additionally incubated with TMB solution (Biolegend, USA) at room temperature for 10 min. Following the addition of 2 M sulfuric acid, the optical density at 450 nm (reference at 570 nm) was measured using a microplate reader (TECAN). The LTA concentrations of LTA-Butanol and LTA-HIC were determined using the regression obtained from optical density of various concentrations of Lp.LTA.

### Thermal, Enzymatic, and Chemical Treatment of LTA and Its Intermediates

To inactivate residual proteins in Lp.LTA and its intermediates, LTA-Butanol, LTA-HIC, and Lp.LTA were treated with proteinase K (50 μg/ml) at 37°C for 1 h as previously described [33]. The Lp.LTA and its intermediates were treated with heat at 100°C for 15 min to inactivate proteins. For removal of both D-alanine and acyl moieties of LTA contained in Lp.LTA and its intermediates, LTA-Butanol, LTA-HIC, and Lp.LTA were incubated in 0.2 N sodium hydroxide for 2 h, and their pH was then adjusted to 7 using 1 N hydrochloric acid [34].

### Crystal Violet Assays

The biofilm biomass was measured by a crystal violet assay as previously described [35]. To examine the effect of Lp.LTA and its intermediates on biofilm formations of oral pathogens, *S. mutans* (1 × 10^6^ CFU/ml) and *P. gingivalis* (1 × 10^8^ CFU/ml), were inoculated in a 96-well plate in the presence or absence of LTA-Butanol, LTA-HIC, or Lp.LTA (0-270 μg/ml). Then, *S. mutans* was cultured at 37°C for 24 h under aerobic and static conditions while *P. gingivalis* was grown at 37°C for 24 h under anaerobic and static conditions. Following the incubation, biofilms were gently rinsed with PBS and stained with 0.1% crystal violet solution at room temperature for 20 min. Biofilms were stained, washed with PBS, and subsequently dissolved in dissociation buffer (95% ethanol and 0.1% acetic acid in distilled water). The optical density at 600 nm was measured using a microplate reader (TECAN).

### Statistical Analysis

Values are expressed as mean values ± standard deviation from triplicates of each treatment group. Statistical significance between non-treatment and indicated Lp.LTA or its intermediate treatment groups was analyzed using the Student’s *t*-test. Asterisks (*) indicate experimental groups that are significantly different (*P* < 0.05) from the designated other group.

## Results

### Concentration of LTA Progressively Increases throughout the Purification Process

**Fig. 1:**
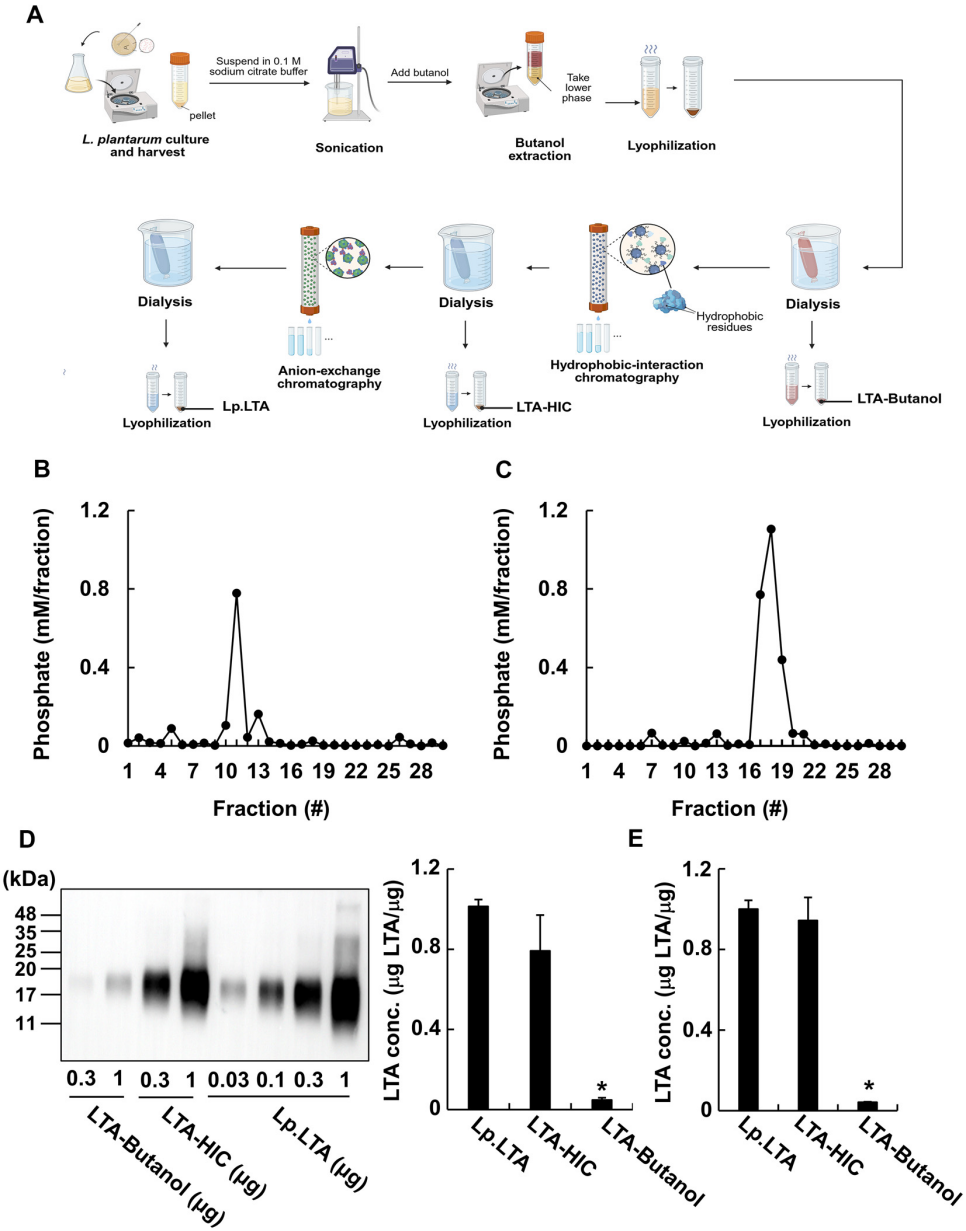
Preparation of Lp.LTA intermediates after each purification process and their LTA concentrations. (**A**) The scheme for preparation of Lp.LTA and its intermediates after each purification process. The LTA-Butanol, LTA-HIC, and Lp.LTA were prepared after butanol extraction, hydrophobic-interaction chromatography, and anion-exchange chromatography, respectively. The quantities were determined by measuring their dry weight. (**B, C**) The inorganic phosphate assays were conducted to measure the quantity of LTA in each fraction after (**B**) hydrophobic-interaction chromatography, and (**C**) anion-exchange chromatography as described in Materials and Methods section. (**D**) The prepared LTA-Butanol, LTAHIC, and Lp.LTA were subjected to Western blotting analysis using an antibody specific for LTA (*left*). The LTA concentrations of LTA-Butanol, LTA-HIC, and Lp.LTA were determined using the regression obtained from the band intensity of various concentrations of Lp.LTA (*right*). (**E**) LTA contents in Lp.LTA and its intermediates were evaluated by ELISA. Data shown are the mean values ± SD of triplicate samples. Asterisk indicates a significant difference between Lp.LTA and other groups at *P* < 0.05. Lp.LTA, LTA purified from *L. plantarum* KCTC 10887BP; LTA-Butanol, Lp.LTA intermediate after butanol extraction; LTA-HIC, Lp.LTA intermediate after hydrophobic-interaction chromatography.

To compare the anti-biofilm capacities of Lp.LTA and its intermediates, LTA-Butanol, and LTA-HIC, we initially prepared them from *L. plantarum* KCTC 10887BP through a serial application of butanol extraction, hydrophobic-interaction chromatography, and anion-exchange chromatography (Fig. 1A). For the butanol extraction, *L. plantarum* lysates were initially mixed with an equal volume of *n*-butanol to extract membrane-bound LTA by disrupting the bacterial phospholipid bilayer. After the centrifugation, lower phase containing LTA was collected for further purification steps thereby removing the insoluble components, such as peptidoglycan, wall teichoic acid (WTA), and cell wall polysaccharides, which remained in the pellet after the centrifugation [36-38]. Then, hydrophobic-interaction chromatography based on differences in hydrophobicity between LTA and other bacterial components was applied to eliminate hydrophobic bacterial components, including membrane lipids, lipoproteins, and hydrophobic proteins [38, 39]. Finally, anion-exchange chromatography was applied to LTA purification to remove residual charged bacterial components, such as endotoxins, nucleic acids, cytoplasmic- and membrane-proteins [40]. During the purification, we conducted an inorganic phosphate assay for every column fraction obtained from both hydrophobic-interaction chromatography and anion-exchange chromatography to collect LTA containing fractions because phosphate contents of column fractions are correlated with their LTA contents [36]. As shown in Fig. 1B and 1C, phosphate-containing fractions after hydrophobic-interaction chromatography were shown in the fractions ranging from 10 to 13, while phosphate was detected in the fractions ranging from 17 to 19 after anion-exchange chromatography. The previously mentioned phosphate-containing column fractions were pooled, dialyzed, and lyophilized to obtain LTA-HIC, and Lp.LTA, respectively. On the other hand, LTA and WTA in *Lactobacillus* are structurally distinct from the other components derived from Gram-positive bacteria by the presence of a poly-Gro-P backbone [20]. However, since WTA is removed by *n*-butanol extraction and hydrophobic-interaction chromatography during LTA purification, Western blot using an antibody specific for Gro-P region of LTA was used for determination of LTA concentrations in Lp.LTA and its intermediates as previously described [41]. Although both LTA-Butanol and LTA-HIC contained LTA, LTA-HIC exhibited relatively higher concentrations of LTA compared to LTA-Butanol (Fig. 1D). Furthermore, the LTA concentrations in Lp.LTA and its intermediates confirmed by ELISA. Similar to the Western blot results, LTA concentration in LTA-HIC was comparable to that in Lp.LTA while LTA level in LTA-Butanol was relatively lower than that of the others (Fig. 1E). In addition, the purity of Lp.LTA and its intermediates was examined by measuring the contents of possible impurities, including proteins, nucleic acids, and endotoxins, that can be introduced during their purification. As shown in Fig. S1A and S1B, the proteins and nucleic acids were rarely detected in both LTA-HIC and Lp.LTA, but not in LTA-Butanol, suggesting that the prepared LTA-HIC and Lp.LTA are highly-pure. In addition, endotoxins were not detected (Data not shown). Collectively, these results suggest that LTA concentration is gradually enhanced throughout its purification steps, and LTA concentration of LTA-HIC was comparable to the Lp.LTA.

### The LTA-HIC Possesses the Anti-Biofilm Activity against Oral Pathogens Comparable to That of Lp.LTA

**Fig. 2:**
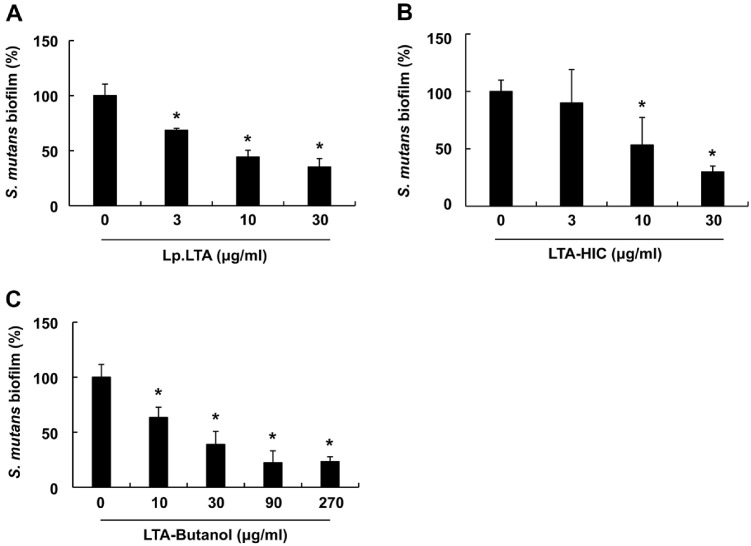
LTA-HIC inhibits *S. mutans* biofilm formation. *S. mutans* (1 × 10^6^ CFU/ml) was inoculated in a 96-well plate in the presence or absence of (**A**) Lp.LTA (0, 3, 10, or 30 μg/ml), (**B**) LTA-HIC (0, 3, 10, or 30 μg/ml), or (**C**) LTA-Butanol (0, 10, 30, 90, or 270 μg/ml) under aerobic and static conditions for 24 h. After the incubation, biofilm biomass was determined by a crystal violet assay as described in the Materials and Methods section. Data shown are the mean values ± SD of triplicate samples. Asterisk indicates a significant difference between non-treatment and treatment groups at *P* < 0.05. Lp.LTA, LTA purified from *L. plantarum* KCTC 10887BP; LTA-Butanol, Lp.LTA intermediate after butanol extraction; LTA-HIC, Lp.LTA intermediate after hydrophobic-interaction chromatography.

**Fig. 3:**
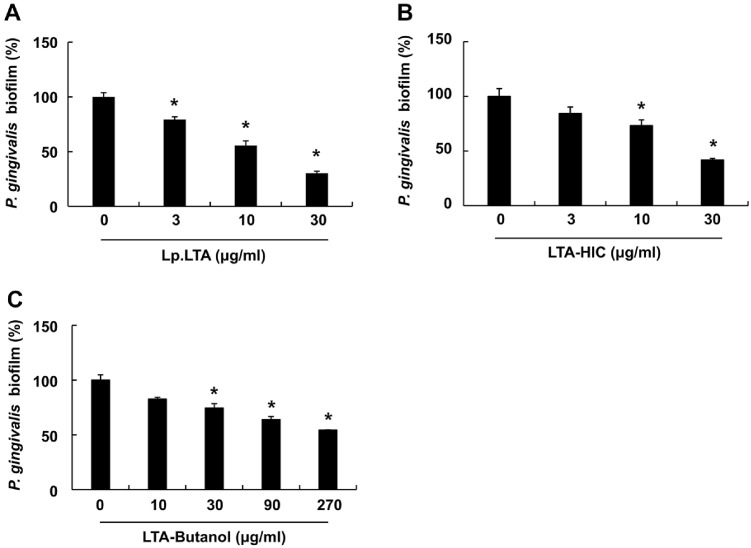
LTA-HIC effectively suppresses the biofilm formation of *P. gingivalis*. *P. gingivalis* (1 × 10^8^ CFU/ml) was cultured in 96-well plate in the presence or absence of (**A**) Lp.LTA (0, 3, 10, or 30 μg/ml), (**B**) LTA-HIC (0, 3, 10, or 30 μg/ml), or (**C**) LTA-Butanol (0, 10, 30, 90, or 270 μg/ml) under anaerobic and static conditions for 24 h. The biofilm biomass was determined by a crystal violet assay. Data shown are the mean values ± SD of triplicate samples. Asterisk indicates a significant difference between non-treatment and treatment groups at *P* < 0.05. Lp.LTA, LTA purified from *L. plantarum* KCTC 10887BP; LTAButanol, Lp.LTA intermediate after butanol extraction; LTA-HIC, Lp.LTA intermediate after hydrophobic-interaction chromatography.

We compared the anti-biofilm activity of the prepared Lp.LTA and its intermediates against oral pathogens, beginning with an evaluation of their efficacy against *S. mutans* using crystal violet staining. As shown in Fig. 2A-2C, all tested Lp.LTA and its intermediates effectively suppressed the *S. mutans* biofilm formation in a dose-dependent manner. Moreover, LTA-HIC showed anti-biofilm activity comparable to that of Lp.LTA at equivalent concentrations (10 and 30 μg/ml). The anti-biofilm capacities of Lp.LTA and its intermediates were also examined against *P. gingivalis*. All Lp.LTA and its intermediates dose-dependently inhibited *P. gingivalis* biofilm formation (Fig. 3A-3C). Notably, LTA-HIC showed comparable anti-biofilm activity to that of Lp.LTA for all tested concentrations, while a relatively higher concentration of LTA-Butanol was required to achieve a similar level of anti-biofilm activity. These results demonstrate that Lp.LTA and its intermediates commonly possess the anti-biofilm properties against both *S. mutans* and *P. gingivalis*, with the anti-biofilm capacity of LTA-HIC being comparable to that of Lp.LTA.

### The Anti-Biofilm Property of LTA-HIC Is Mainly Mediated by LTA

**Fig. 4:**
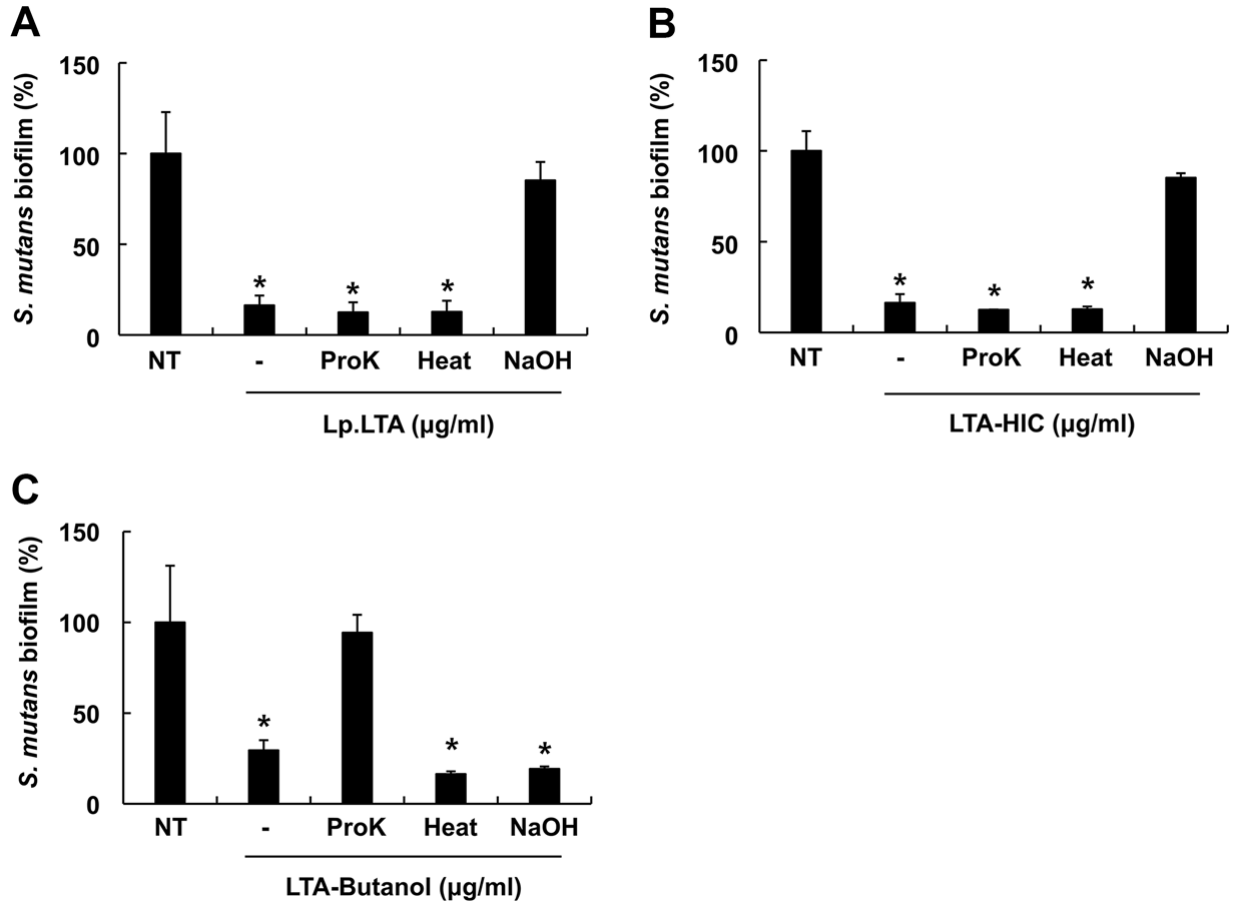
The anti-biofilm activity of LTA-HIC against *S. mutans* biofilm formation is mediated by LTA. *S. mutans* (1 × 10^6^ CFU/ml) was grown in 96-well plate in the presence or absence of (**A**) 30 μg/ml of Lp.LTA, proteinase K-treated Lp.LTA, heat-treated Lp.LTA or sodium hydroxide-treated Lp.LTA, (**B**) 30 μg/ml of LTA-HIC, proteinase K-treated LTA-HIC, heattreated LTA-HIC or sodium hydroxide-treated LTA-HIC, or (**C**) 270 μg/ml of LTA-Butanol, proteinase K-treated LTA-Butanol, heat-treated LTA-Butanol, or sodium hydroxide-treated LTA-Butanol under anaerobic and static conditions for 24 h. After washing with PBS, remaining biofilms were determined by a crystal violet assay. Data shown are the mean values ± SD of triplicate samples. Asterisk indicates a significant difference between non-treatment and treatment groups at *P* < 0.05. NT, nontreatment; Lp.LTA, LTA purified from *L. plantarum* KCTC 10887BP; LTA-Butanol, Lp.LTA intermediate after butanol extraction; LTA-HIC, Lp.LTA intermediate after hydrophobic-interaction chromatography; ProK, proteinase K treated; Heat, heat treated; NaOH, sodium hydroxide treated.

**Fig. 5:**
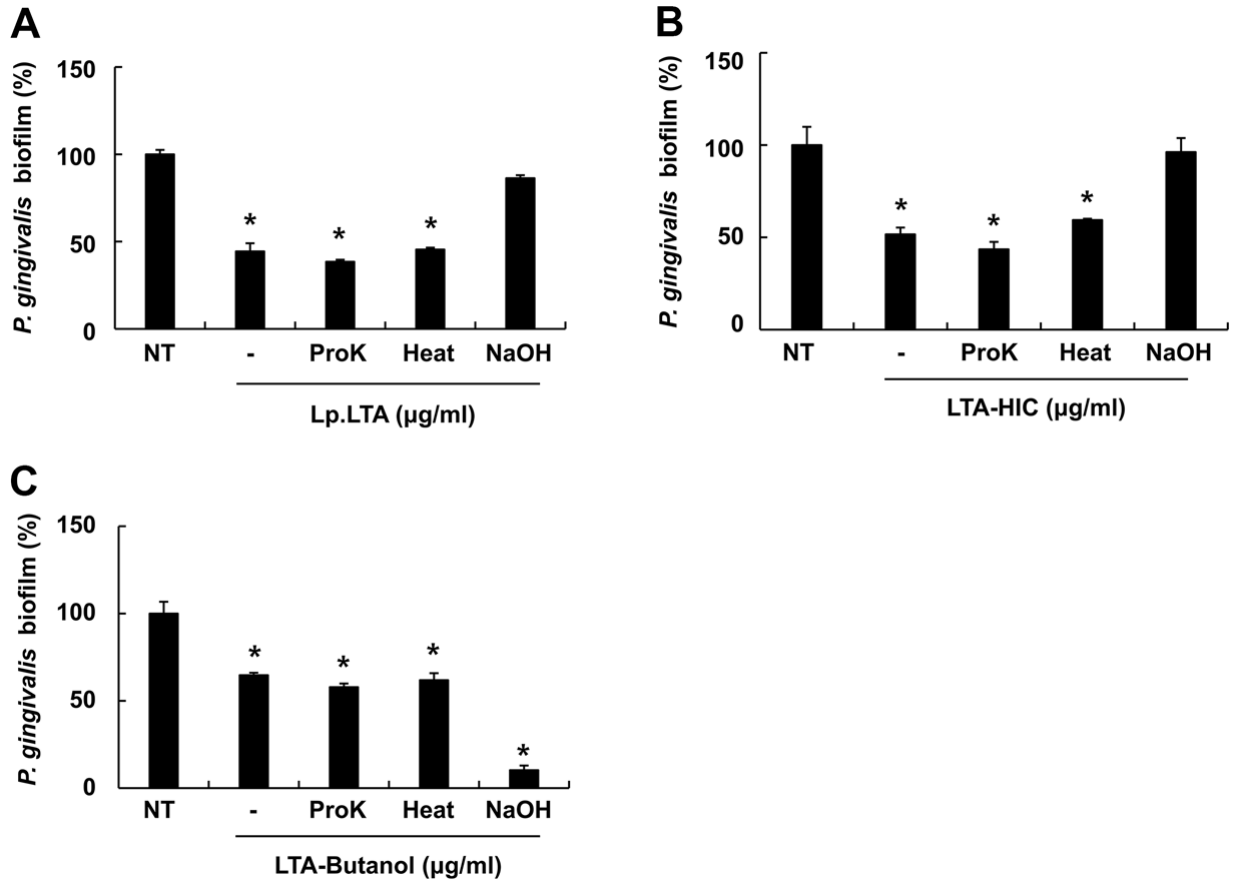
LTA is a key molecule responsible for the anti-biofilm activity of LTA-HIC against *P. gingivalis* biofilm formation. *P. gingivalis* (1 × 10^8^ CFU/ml) was cultured in 96-well plate in the presence or absence of (**A**) 30 μg/ml of Lp.LTA, proteinase K-treated Lp.LTA, heat-treated Lp.LTA or sodium hydroxide-treated Lp.LTA, (**B**) 30 μg/ml of LTA-HIC, proteinase K-treated LTA-HIC, heat-treated LTA-HIC or sodium hydroxide-treated LTA-HIC, or (**C**) 270 μg/ml of LTA-Butanol, proteinase K-treated LTA-Butanol, heat-treated LTA-Butanol, or sodium hydroxide-treated LTA-Butanol under anaerobic and static conditions for 24 h. The biofilm biomass was then determined by a crystal violet assay. Data shown are the mean values ± SD of triplicate samples. Asterisk indicates a significant difference between non-treatment and treatment groups at *P* < 0.05. NT, non-treatment; Lp.LTA, LTA purified from *L. plantarum* KCTC 10887BP; LTA-Butanol, Lp.LTA intermediate after butanol extraction; LTA-HIC, Lp.LTA intermediate after hydrophobic-interaction chromatography; ProK, proteinase K treated; Heat, heat treated; NaOH, sodium hydroxide treated.

To identify the molecules responsible for the anti-biofilm capacities of LTA-Butanol and LTA-HIC, we examined the effects of Lp.LTA and its intermediates on *S. mutans* and *P. gingivalis* biofilm formation after treating them with proteinase K, heat, or sodium hydroxide. In addition, the heat and proteinase K was used to inactivate proteins and enzymes, respectively. Furthermore, sodium hydroxide was used to remove acyl chains and D-alanine residues of LTA thereby inactivating biological activities of LTA [42]. Fig. 4A and 4B demonstrated that treatment with proteinase K and heat did not affect their inhibitory effects of Lp.LTA and LTA-HIC on *S. mutans* biofilm formation. In contrast, sodium hydroxide treatment decreased their anti-biofilm activities against *S. mutans*. The anti-biofilm capacity of LTA-Butanol was restored by proteinase K treatment, but not by heat or sodium hydroxide treatments (Fig. 4C). Meanwhile, we also examined how the previously mentioned treatments on Lp.LTA and its intermediates affect biofilm formation of *P. gingivalis*. Similar to the results on *S. mutans* biofilm formation, the anti-biofilm activities of Lp.LTA and LTA-HIC against *P. gingivalis* were also diminished by sodium hydroxide treatment, but not by proteinase K and heat treatments (Fig. 5A and 5B). Interestingly, the inhibitory effect of LTA-Butanol on *P. gingivalis* biofilm formation remained uninfluenced by proteinase K, and heat treatments. However, biofilm formation was significantly reduced with sodium hydroxide treated LTA-Butanol compared to untreated LTA-Butanol (Fig. 5C). Based on the results that both Lp.LTA and LTA-HIC contained high concentrations of LTA (Fig. 1D and 1E) with negligible levels of impurities (Fig. S1A and S1B), these results suggest that LTA is a key component responsible for the inhibitory effect of LTA-HIC on oral pathogen biofilm formation. On the other hand, the inhibitory effect of LTA-Butanol might be resulted from its contained *L. plantarum* derived biomolecules rather than LTA.

## Discussion

The current study demonstrated that both Lp.LTA and its intermediates, specifically LTA-Butanol and LTA-HIC, effectively inhibited biofilm formation of *S. mutans* and *P. gingivalis* following each purification process. Moreover, the anti-biofilm activity of LTA-HIC against the biofilm formation of the dental pathogens was more potent than that of LTA-Butanol and comparable to Lp.LTA. Furthermore, sodium hydroxide treatment of LTA-HIC for the removal of both D-alanine and acyl moieties of LTA reduced its anti-biofilm activities. This suggests that LTA is a major component of LTA-HIC responsible for its anti-biofilm activity against *S. mutans* and *P. gingivalis*.

In this study, we found that the anti-biofilm capacity of LTA-HIC is comparable to that of Lp.LTA and the property was further mediated by LTA. The practical application of LTA-HIC as an anti-biofilm reagent instead of LTA might have several advantages. First, purification of LTA-HIC is more time- and labor-effective than LTA isolation. In fact, purification of LTA-HIC takes less than 21 days, whereas Lp.LTA isolation takes over 28 days of time. Second, since the purification yield of LTA-HIC is 2.1 fold higher than that of Lp.LTA (Table S1), its application is considered to have better economic efficiency than Lp.LTA. Third, since LTA-HIC isolation is relatively simpler than that of LTA, it can help minimize large-scale LTA purification processes for its practical application. Therefore, LTA-HIC might be a more practical form of Lp.LTA as an anti-biofilm agent for controlling the infection of dental pathogens, overcoming the limitations in a Lp.LTA purification process.

In the current study, we demonstrated that the anti-biofilm capacity of LTA-HIC is mainly mediated by the presence of LTA. Although we previously reported various underlying mechanisms responsible for the anti-biofilm property of Lp.LTA against *S. mutans* [24], further studies are required to characterize the underlying mechanisms for *P. gingivalis*. Based on our previous studies about Lp.LTA, the anti-biofilm property of Lp.LTA on *P. gingivalis* could be explained by several proposed mechanisms. First, Lp.LTA inhibits initial surface adhesion by direct interaction with *P. gingivalis*. This potential mechanism is supported by our previous studies, which demonstrated that the surface attachment of *S. mutans* was blocked on Lp.LTA pre-coated plate [33], and that Lp.LTA exerted an inhibitory effect on biofilm formation of *E. faecalis*, *S. mutans*, and *Staphylococcus aureus* at an early stage within 3 h of incubation [24, 25, 33]. Second, Lp.LTA in LTA-HIC might be involved in the regulation of a representative quorum molecule, autoinducer-2 (AI-2), thereby exerting its anti-biofilm property on *P. gingivalis*. In our previous study, we reported that enhanced AI-2 release by Lp.LTA inhibited *S. aureus* biofilm formation by suppressing the intracellular adhesion (*ica*) gene expression, which is essential for production of polysaccharide intercellular adhesion [33]. Moreover, since *P. gingivalis* possesses the AI-2-mediated quorum-sensing system [43], regulation of quorum-sensing signaling by Lp.LTA might be another potential mechanism. The previously mentioned mechanisms should be verified through further studies.

Like the LTA-HIC and Lp.LTA, LTA-Butanol also inhibited the biofilm formation of *S. mutans* and *P. gingivalis*. However, since the anti-biofilm property of LTA-Butanol was not eliminated by its sodium hydroxide treatment, the capacity is likely to be caused by other *L. plantarum*-derived biomolecules rather than LTA. Moreover, inhibited *S. mutans* biofilm formation by LTA-Butanol was restored by proteinase K treatment, but not by heat and sodium hydroxide treatments, suggesting a possibility that the anti-biofilm capacity of LTA-Butanol, at least against *S. mutans*, is supposedly mediated by *L. plantarum*-derived heat stable proteins. Furthermore, the aforementioned possibility is supported by the results from BCA protein assay, which showed relatively higher concentrations of proteins in LTA-Butanol compared to those in LTA-HIC and Lp.LTA (Fig. S1A). According to previous studies, *L. plantarum* produces bacteriocins, particularly the plantaricin family, which exhibit anti-biofilm activity and effectively suppress biofilm formation of Gram-positive pathogens, such as *E. faecalis* and *Listeria monocytogenes* [44, 45]. These proteins are typically heat stable and hydrophobic [46], and they lack phosphate in their structure. Consequently, they can be incorporated into LTA-Butanol, but not into LTA-HIC, which is derived from the hydrophobic fractions containing phosphate after the inorganic phosphate assay. Therefore, the anti-biofilm capacity of LTA-Butanol against *S. mutans* is likely attributed to the plantaricins present in LTA-Butanol rather than to LTA itself. On the other hand, since it has been reported that most of plantaricins are rarely effective against Gram-negative bacteria due to the structural differences in the cell membrane between Gram-negative and Gram-positive bacteria [47], suppressed *P. gingivalis* biofilm formation by LTA-Butanol does not appear to be restored by proteinase K treatment.

Although the anti-biofilm capacity of LTA-Butanol against *P. gingivalis* was enhanced by its sodium hydroxide treatment, the treatment did not affect the anti-biofilm capacity of LTA-Butanol against *S. mutans*. Based on our results that the anti-biofilm property of LTA-Butanol was relatively higher in *S. mutans* than *P. gingivalis*, LTA-Butanol may contain *L. plantarum*-derived biomolecules which promotes the biofilm formation of *P. gingivalis*, but not that of *S. mutans*. According to previous studies, LTA-Butanol, which is obtained from lower phase after the butanol extraction, may contain various *L. plantarum* derived biomolecules, such as LTA, hydrophilic proteins, EPS, and nucleic acids [48-50]. Among them, extracellular DNA (eDNA) is known to be involved in bacterial biofilm formation by acting as a key structural component of EPS and enhancing bacterial adhesion to surface and cell-cell aggregation [51, 52]. Furthermore, exogenous eDNA can also be involved in bacterial biofilm formation and its effects seem to be different depending on bacterial species. According to a previous study, externally supplemented eDNA showed limited effect on both bacterial adherence and biofilm formation of *S. mutans* [53]. In contrast, the addition of exogenous eDNA enhanced the biofilm formation of various Gram-negative bacteria, such as *Acinetobacter baumannii*, *Pseudomonas aeruginosa*, and *Klebsiella pneumoniae* [54, 55]. These observations suggest a possibility that eDNA contained in LTA-Butanol could enhance the biofilm formation of *P. gingivalis*, but not that of *S. mutans*. Furthermore, the inactivation of eDNA in LTA-Butanol by sodium hydroxide might cause the more reduced biofilm formation of *P. gingivalis* compared to untreated LTA-Butanol. This possibility is supported by the results from spectrophotometic analysis that LTA-Butanol contained relatively higher concentrations of nucleic acids compared to those LTA-HIC and Lp.LTA (Fig. S1B).

Since currently available dental medicaments do not sufficiently eradicate dental pathogen biofilms, alternative strategies for controlling the biofilm are required. In our previous studies, we demonstrated that *Lactobacillus* LTAs possessed potent anti-biofilm properties against various dental pathogens, including *S. mutans*, *S. gordonii*, and *E. faecalis* [24-27]. In addition, Lp.LTA has the most potent anti-biofilm activity among the tested *Lactobacillus* LTAs [24, 25]. Nevertheless, practical application of Lp.LTA as an anti-biofilm agent is limited due to its relatively complex, cost- and labor-intensive purification process, as well as its low purification yield, suggesting the need for a more efficient and low-cost LTA purification process. In the current study, we proved that LTA concentration and anti-biofilm activity of LTA-HIC are comparable to Lp.LTA, suggesting that LTA-HIC could be applied as a practical anti-biofilm agent instead of Lp.LTA for treatment of dental caries and periodontitis. On the other hand, since we previously reported that *Lactobacillus* LTA pretreatment could feasibly be used with various dental disinfectants for treatment of dental pathogens [26, 35], LTA-HIC may also be utilized as a supplementary medicament for treating dental diseases, such as dental caries and periodontitis, and this possibility should be evaluated through further study.

## Supplemental Materials

Supplementary data for this paper are available on-line only at http://jmb.or.kr.
