# Supplementary material for: Optimization of the Purification Process for Lactiplantibacillus plantarum Lipoteichoic Acid with Anti-Biofilm Properties against Dental Pathogens
Source: J Microbiol Biotechnol. 2025 Sep 22;35:e2506045. doi: 10.4014/jmb.2506.06045 (PMC12535855; doi:10.4014/jmb.2506.06045)
Supplement: Supplementary file 1 [file jmb-35-e2506045-supple.pdf]

J. Microbiol. Biotechnol. 2025. 35: e2506045

https://doi.org/10.4014/jmb.2506.06045

Supplementary Table

Table S1. Purification yield of Lp.LTA and its intermediates.

	Bacterial pellet (g wet weight)	Purification yield (mg dry weight)	Purification yield/g pellet (mg dry weight/g pellet)
LTA-Butanol[a]	86	383.37	4.46
LTA-HIC[b]	86	24.56	0.29
Lp.LTA[c]	86	12.12	0.14

[a] LTA-Butanol, Lp.LTA intermediate after butanol extraction; [b] LTA-HIC, Lp.LTA intermediate after hydrophobic-interaction chromatography; [c] Lp.LTA, LTA purified from *L. plantarum* KCTC 10887BP
